# Epidural analgesia during labour and severe maternal morbidity: population based study

**DOI:** 10.1136/bmj-2023-077190

**Published:** 2024-05-22

**Authors:** Rachel J Kearns, Aizhan Kyzayeva, Lucy O E Halliday, Deborah A Lawlor, Martin Shaw, Scott M Nelson

**Affiliations:** 1 Department of Anaesthesia, Glasgow Royal Infirmary, Glasgow, UK; 2 School of Medicine, University of Glasgow, Glasgow Royal Infirmary, Glasgow, G31 2ER, UK; 3 MRC Integrative Epidemiology Unit at the University of Bristol, Bristol, UK; 4 Population Health Science, University of Bristol, Bristol, UK; 5 Department of Medical Physics and Bioengineering, NHS Greater Glasgow and Clyde, Glasgow, UK

## Abstract

**Objectives:**

To determine the effect of labour epidural on severe maternal morbidity (SMM) and to explore whether this effect might be greater in women with a medical indication for epidural analgesia during labour, or with preterm labour.

**Design:**

Population based study.

**Setting:**

All NHS hospitals in Scotland.

**Participants:**

567 216 women in labour at 24+0 to 42+6 weeks’ gestation between 1 January 2007 and 31 December 2019, delivering vaginally or through unplanned caesarean section.

**Main outcome measures:**

The primary outcome was SMM, defined as the presence of ≥1 of 21 conditions used by the US Centers for Disease Control and Prevention (CDC) as criteria for SMM, or a critical care admission, with either occurring at any point from date of delivery to 42 days post partum (described as SMM). Secondary outcomes included a composite of ≥1 of the 21 CDC conditions and critical care admission (SMM plus critical care admission), and respiratory morbidity.

**Results:**

Of the 567 216 women, 125 024 (22.0%) had epidural analgesia during labour. SMM occurred in 2412 women (4.3 per 1000 births, 95% confidence interval (CI) 4.1 to 4.4). Epidural analgesia was associated with a reduction in SMM (adjusted relative risk 0.65, 95% CI 0.50 to 0.85), SMM plus critical care admission (0.46, 0.29 to 0.73), and respiratory morbidity (0.42, 0.16 to 1.15), although the last of these was underpowered and had wide confidence intervals. Greater risk reductions in SMM were detected among women with a medical indication for epidural analgesia (0.50, 0.34 to 0.72) compared with those with no such indication (0.67, 0.43 to 1.03; P<0.001 for difference). More marked reductions in SMM were seen in women delivering preterm (0.53, 0.37 to 0.76) compared with those delivering at term or post term (1.09, 0.98 to 1.21; P<0.001 for difference). The observed reduced risk of SMM with epidural analgesia was increasingly noticeable as gestational age at birth decreased in the whole cohort, and in women with a medical indication for epidural analgesia.

**Conclusion:**

Epidural analgesia during labour was associated with a 35% reduction in SMM, and showed a more pronounced effect in women with medical indications for epidural analgesia and with preterm births. Expanding access to epidural analgesia for all women during labour, and particularly for those at greatest risk, could improve maternal health.

## Introduction

The rising incidence of severe maternal morbidity (SMM) constitutes a pressing global issue, compromising the wellbeing of mothers and their children, and resulting in potentially devastating short term and long term consequences.^1^ ^2^ SMM is defined by the US Centers for Disease Control and Prevention (CDC) as encompassing 21 indicative conditions or procedures, such as myocardial infarction, eclampsia, and hysterectomy occurring during admission to hospital for delivery.^3^ In the UK, the incidence of SMM almost doubled between 2009 and 2018, from 0.9% to 1.7% of deliveries, likely reflecting the trend of mothers being older, more obese, and with increasing comorbidities, along with a rising incidence of previous caesarean delivery.^4^ SMM can be conceptualised as an indicator of increased risk for maternal mortality, providing crucial opportunities to identify and implement interventions to improve the health of mothers and their offspring.^5^

Epidural analgesia is commonly advised for safety reasons in pregnant women considered at higher risk of SMM, such as those with multiple births, morbid obesity (body mass index (BMI) ≥40), or certain comorbidities, owing to its advantageous physiological effects and capacity to provide expedient anaesthesia if required in an emergency.^6^ Women with these factors can be considered as having a medical indication for epidural analgesia during labour. Women giving birth preterm also carry a higher risk of SMM, although epidural analgesia is seldom recommended for preterm labour alone.^7^ Despite the assumed benefits of epidural analgesia during labour to prevent SMM, the evidence base for this is limited. We identified just two observational studies that attempted to delineate the association between epidural analgesia during labour and SMM.^8^ ^9^ One, a US study (n=574 525), indicated a 14% risk reduction in SMM in women who received epidural analgesia, but it only included vaginal births and excluded the six week postnatal period, during which about 15% of SMM events occur.^8^ ^10^ The other study, from France (n=4550), reported a 47% decreased risk of severe postpartum haemorrhage in women with epidural analgesia who gave birth vaginally, but it did not assess other constituents of SMM.^9^ Neither of these studies explored whether the association differed between women with a medical indication and those without, or between women who delivered preterm and those who did not. In these two studies from countries with private healthcare systems, the use of epidural analgesia was 47%^8^ and 78%,^9^ respectively, whereas in the UK, the use of epidural analgesia during labour is around 22-30%, despite healthcare being free at the point of access.^11^ ^12^

Notwithstanding that clinicians may advise mothers with medical indications about epidural analgesia during labour, the final decision is up to the woman. The lack of robust evidence on whether benefits exist beyond the provision of epidural analgesia might affect the discussions clinicians have with women and their decisions. Women from minority ethnic groups and areas of socioeconomic deprivation are at higher risk of maternal morbidity and mortality, and they are more likely to have medical indications for epidural analgesia, but are less likely to have one.^13^ ^14^ ^15^ Stronger evidence on the effects of epidural analgesia might contribute to reducing these inequalities. The importance of improving this evidence base is highlighted by the priority setting exercises undertaken by the James Lind Alliance, which identified the effect of epidural analgesia on obstetric outcomes as a research priority.^16^ The James Lind Alliance brings patients, carers, and clinicians together to identify research priorities.

In this population based cohort analysis of all births in Scotland over a 13 year period, we estimated the causal effect of the use of epidural analgesia during labour on SMM in all mothers, except those undergoing planned caesarean section delivery. Additionally, we explored whether this effect was more pronounced among pregnant women who according to clinical guidelines are at increased risk of SMM (ie, women with a medical indication for epidural analgesia during labour), and in those with preterm labour.

## Methods

Our methods are reported in accordance with Strengthening the Reporting of Observational Studies in Epidemiology (STROBE) guidance.^17^

### Data sources and study population

We linked six Scotland-wide administrative databases: the Scottish Morbidity Record-2 (SMR02), the Scottish Morbidity Record-1 (SMR01), the Scottish Birth Record, the National Records of Scotland, the Scottish Stillbirth Infant Death Survey, and the Scottish Intensive Care Society Audit Group. The SMR02 documents all obstetric inpatient and day case admissions during pregnancy and the postnatal period and includes maternal and infant characteristics. The SMR02 is subject to regular quality assurance checks, with data more than 99% complete since the late 1970s.^18^ ^19^ The SMR01 records all non-obstetric inpatient and day case admissions according to ICD-9 and ICD-10 (international classification of diseases, ninth revision and 10th revision, respectively) codes and UK NHS OPCS-4 (Office of Population Censuses and Surveys classification of interventions and procedures).^20^ ^21^ All neonatal care is recorded in the Scottish Birth Record. The National Records of Scotland registers all births, stillbirths, and infant deaths, and the Scottish Stillbirth Infant Death Survey collects additional information from the relevant coordinator of the survey (obstetrician, paediatrician, or midwife) at each hospital. The database of the Scottish Intensive Care Society Audit Group records admission data for all Scottish intensive care and high dependency units, with regular data validation.^22^

### Inclusion and exclusion criteria

We analysed all women in labour in Scotland between 1 January 2007 and 31 December 2019 with gestation between 24+0 and 42+6 weeks. Births were excluded after this period to remove any potential confounding influence of the covid-19 pandemic. We also excluded births when mode of delivery, child identity, or data for analgesia during labour were not recorded (n=38 705, 5.5% of all 697 981 pregnancies considered); see supplementary eFigure 1), as well as births by elective caesarean section as these women knew their mode of delivery in advance and would not experience labour, and therefore by definition could not have chosen to have epidural analgesia (n=92 060, 13.2%; see supplementary eFigure 1).

### Epidural analgesia

We defined epidural analgesia during labour as conventional lumbar epidural sited at any time during labour. This definition is consistent with standard medical practices in the UK, where epidural drugs are generally administered only after labour has commenced. We were unable to identify use of combined spinal epidural (spinal injection plus insertion of an epidural catheter), as SMR02 classifies the procedure as spinal anaesthesia. Combined spinal epidural is used infrequently in Scotland, representing only 1% of epidural use during labour.^23^ Women recorded as having no epidural could have delivered without additional analgesia or anaesthesia or have required spinal or general anaesthesia for operative delivery, reflecting the unpredictability of labour outcomes and the resultant different potential pathways care may take. Since recording of anaesthetic intervention is hierarchical, we could not identify if women who had a spinal or general anaesthetic also had epidural analgesia at an earlier point. Conversion of epidural analgesia to spinal or general anaesthesia occurs in around 5% of women.^24^

### Outcomes

The primary outcome was SMM, defined as a composite outcome of ≥1 of 21 conditions according to the US CDC criteria for SMM or a critical care admission, with either occurring at any point from the date of delivery to 42 days post partum (described as SMM). In keeping with other published data, we incorporated critical care admission as an SMM indicator because the CDC’s definition does not cover all SMM events (eg, asthma attack, status epilepticus).^4^ We identified conditions using ICD-9, ICD-10, and OPCS codes from SMR01, SMR02, and Scottish Intensive Care Society Audit Group datasets (see supplementary eTable 1 for table of codes).^3^ The CDC’s definition of SMM has a sensitivity of 77% and specificity of 99% in identifying SMM compared with medical records.^25^

Secondary outcomes aimed to capture more severe morbidity and included ≥1 of the 21 CDC conditions when that condition resulted in admission for critical care (described as SMM plus critical care), and respiratory morbidity (ventilation, tracheostomy, acute respiratory distress syndrome, or respiratory complications of anaesthesia), as diagnosed from the date of delivery to 42 days post partum (see supplementary eTable 1).

Minor modifications were made to the CDC SMM criteria to accommodate data recording practices in Scotland (see supplementary eTable 1). In line with other UK studies,^4^ ^26^ we found that the UK definition for postpartum haemorrhage (≥500 mL blood loss) resulted in over-reporting of major obstetric haemorrhage (ICD-10 code O72), and therefore we included postpartum haemorrhage only if it occurred in association with a critical care admission, indicating a clinically significant haemorrhage event. Alternative metrics such as volume of blood loss and blood transfusion are not reliably recorded in SMR02. Similar to a previous Scottish study, we found the incidence of sepsis had increased exponentially from 2012 (see supplementary eFigure 2).^4^ This might reflect different coding practices and changes in guidance with the publication of the 2012 Surviving Sepsis recommendations resulting in increased awareness of the condition.^27^ ^28^ Because sepsis is defined as the presence of an infection and evidence of acute organ dysfunction, we included it only if associated with admission to a critical care unit.

Given that in our analyses, as in any risk analyses, we censored at the first SMM condition, the difference between the primary outcome and the first secondary outcome is illustrated by considering a mother with eclampsia diagnosed on the day of delivery and acute heart failure diagnosed on postnatal day 22. In the primary analysis, that woman would be censored on the day of delivery. In contrast, a woman with eclampsia diagnosed on the day of delivery who experienced heart failure resulting in critical care admission at 22 days postnatally when heart failure was diagnosed, would be censored at postnatal day 22. Conversely, a woman with the same conditions at the same time points but who was not admitted to critical care for either would not be considered at risk for the secondary outcome of SMM plus critical care admission (and would contribute to the comparator group—no SMM plus critical care).

### Confounders and other variables used in analyses

To determine confounding variables before analyses, we used the established definition of a confounder—something that is a known or plausible reason for having both epidural analgesia during labour and SMM, and we considered all potential plausible pathways between these variables.^29^ We included these confounders (irrespective of whether they were available in our data) in directed acyclic graphs drawn using the R package “DAGGitty,”^30^ to highlight sources of unmeasured confounding and how these might be captured by other measured confounders on the same confounding path (see supplementary eFigures 3a and 3b). We included socioeconomic status and ethnicity as these factors are increasingly recognised as influencing poor maternal outcomes and epidural analgesia use during labour.^13^ ^14^ ^15^ Ethnicity was defined using NHS Scotland 2011 census categories.^31^ As we did not have information on individual socioeconomic status, we used residential area deprivation according to the Scottish index for multiple deprivation as a proxy; the first 10% of deprivation denoting the most deprived areas and the last 10% the least deprived.^32^ Pre-existing comorbidities that plausibly influence the use of epidural analgesia and SMM were defined for each mother by calculating a Bateman index score, an extensively validated, weighted, risk prediction tool including 20 conditions plus maternal age that is specific to obstetric patients and more accurately predicts SMM than other generic comorbidity indices (see supplementary eTable 2)^33^ To avoid conflating comorbid conditions with the outcome of SMM, we applied strict criteria, restricting these diagnoses to the period between 180 days before the estimated date of conception (as described in the original paper by Bateman et al)^33^ and the day before delivery. This approach ensured the validity of our findings by accurately reflecting the impact of comorbidities on risk of SMM. Using ICD-9 and ICD-10 codes from SMR02, we obtained information on maternal height, weight, and smoking status plus obstetric indices of previous caesarean section, parity, and induction of labour. Gestational age at birth was based on ultrasound assessment in the first half of pregnancy. Smoking status at booking was defined as current, former, or never. Birth location was categorised into obstetric unit, freestanding midwifery unit, or home birth. Obstetric units were defined as hospitals with on-site obstetric and anaesthetic services, inclusive of epidural analgesia provision, or midwifery led units co-located with an obstetric unit. Freestanding midwifery units were defined as midwifery led units without direct access to obstetric or anaesthetic services.^34^

In exploratory analyses we assessed whether associations differed by the presence of a medical indication for epidural analgesia and by gestational age. We classified births as preterm if they occurred before 37 weeks’ gestation and as term or post term if they occurred at ≥37+0 weeks. Births were further classified using World Health Organization (WHO) criteria as extremely preterm (<28 weeks), very preterm (28 to <32 weeks), and moderate to late preterm (≥32 to 36+6 weeks), and by whether labour occurred spontaneously or was commenced iatrogenically.^35^

We defined medical indications for epidural analgesia as any of serious cardiovascular or respiratory disease (congestive heart failure, congenital heart disease, pulmonary hypertension, ischaemic heart disease, asthma); pre-eclampsia; previous caesarean section; breech presentation; multiple pregnancy; and morbid obesity (BMI ≥40), diagnosed before the date of delivery and with no contraindication to epidural insertion (see supplementary eTable 3).^6^ ^36^ ^37^ ^38^ ^39^ ^40^ ^41^ These indications are easily identified by obstetric, anaesthesia, and midwifery staff, reflect criteria that drive common decision making processes, and are in widespread use in clinical practice. These conditions were included if recorded up to the day pre-delivery to ensure they occurred before the decision to have an epidural and any episodes of SMM.

### Statistical analysis

As this was a whole population study, we did not perform sample size calculations. We report baseline characteristics by epidural status. Continuous variables are expressed as medians with interquartile range (IQR), and categorical variables as counts and percentages. For group comparison, we used standardised differences.

To adjust for confounders, we used multivariable Poisson regression models with cluster robust sandwich estimators under the generalised estimation equation framework (see supplementary eFigures 3a and 3b). These models were chosen in place of log-binomial models to avoid problems with convergence. The robust estimator was used to correct the inflated variance found from the standard Poisson model, and to account for more than one birth in some women.^42^ We also assessed a zero inflated Poisson model using a single zero inflation parameter applied to all observations to account for any excess of zeros in the model. This indicated no excess of zeros (P>0.9), further supporting the use of a multivariable Poisson regression model with cluster robust errors. In the modelling of risk analyses, we censored at the first SMM condition (ie, a mother with two SMM conditions was only counted once in the analysis). These models were used to determine adjusted relative risks and absolute risks. As we a priori assumed that outcomes might differ depending on gestational age, we included this as an interaction and adjusted for all of the other previously defined confounders. To explore potential residual confounding from confounders that we did not consider because evidence was lacking to suggest they would affect epidural use and SMM, we calculated an E-value.^43^ The E-value was defined as the minimum strength of association that one confounder or several unmeasured confounders would need to have with both epidural analgesia and SMM, conditional on the confounders we adjusted for, to fully explain a specific exposure-outcome association. This was calculated using the EValue package (version 4.1.3).

#### Exploratory subgroup analyses

We repeated the same adjusted Poison regression modelling cluster robust sandwich estimators as described for the main analyses in three sets of subgroup analyses: Women with a medical indication and those without a medical indication, women delivering pre-term (<37 completed weeks of gestation) and those delivering at term or post term (≥37 completed weeks), and women with a medical indication and delivering preterm and those with no medical indication and delivering at term or post term.

In each of these analyses we tested statistical evidence for a difference between the two related subgroups by comparing a model with an interaction term (eg, interaction term between epidural analgesia during labour and medical indication—yes *v* no) using a likelihood ratio test comparing these two models. As analyses between subgroups are often under-powered, we considered a P value <0.01 to provide statistical evidence of a difference.

As our definition of medical indication for epidural analgesia included some components of the Bateman index score and BMI, we removed Bateman index score and maternal height and weight as confounding variables in the models of subgroup analyses that included medical indication (see supplementary eFigure 3b). Finally, to further model the effect of epidural analgesia on women with different underlying risk profiles for SMM, we analysed the association between epidural analgesia and SMM in women with and without an indication for epidural throughout the continuum of gestational ages using robust Poisson regression with non-linear splines.

#### Additional analyses

Given that epidural analgesia is only available to women delivering in an obstetric unit, we repeated the analyses restricted to births occurring within an obstetric unit (n=541 389, 95.4% of eligible women) and compared the results to our main analyses. We also provided additional subgroup analyses using WHO criteria of preterm births, and by iatrogenic or spontaneous preterm birth.^35^

#### Dealing with missing confounder data

All eligible women (see supplementary eFigure 1) had complete data on epidural analgesia and outcome. Missing data on confounders varied, with the least for maternal age (0 missing) and most for maternal ethnicity (n=222 213, 39.2%) and illicit drug use (n=179 284, 31.6%) (table 1). In total, 257 713 (45.4%) of eligible participants had missing data on ≥1 confounders. We imputed missing data for confounders using multiple imputations through chained equations to form 10 imputed datasets employing a predictive mean matching methodology.^44^ Ten iterations assured data output stability, and 10 imputations guaranteed the accuracy of pooled variable effect size estimates.

**Table 1:** Maternal and neonatal characteristics of pregnant women after exclusion of data missing for epidural analgesia during labour. Values are number (percentage) unless stated otherwise

Characteristics	Overall (n=567 216)	No epidural analgesia (n=442 192)	Epidural analgesia (n=125 024)	Standardised difference (95% CI)
Median (IQR) maternal age (years)	29.0 (25.0-33.0)	29.0 (25.0-33.0)	29.0 (24.0-33.0)	0.37 (0.34-0.41)
Median (IQR) maternal weight (kg)	67.0 (59.0-79.0)	67.0 (59.0-79.0)	68.0 (60.0-80.0)	−1.1 (−1.2 to −1.0)
Missing data	53 692 (9.5)	42 581 (9.6)	11 111 (8.9)	
Median (IQR) maternal height (cm)	164.0 (160.0-169.0)	165.0 (160.0-169.0)	164.0 (160.0-168.0)	0.37 (0.33 to 0.41)
Missing data	60 005 (10.6)	47 561 (10.7)	12 444 (10.0)	
Median (IQR) maternal BMI	24.8 (22.0-28.9)	24.7 (22.0-28.7)	25.1 (22.3-29.4)	−0.50 (−0.54 to −0.46)
Missing data	68 814 (12.1)	54 647 (12.4)	14 167 (11.3)	
Ethnic group:				
White	319 496.0 (92.6)	244 987.0 (92.6)	74 509.0 (92.5)	0.09 (−0.12 to 0.30)
Black	5232.0 (1.5)	4119.0 (1.6)	1113.0 (1.4)	0.18 (0.08 to 0.27)
Mixed	1638.0 (0.5)	1208.0 (0.5)	430.0 (0.5)	−0.08 (−0.13 to −0.02)
Other	3370.0 (1.0)	2559.0 (1.0)	811.0 (1.0)	−0.04 (−0.12 to 0.04)
Asian	15 267.0 (4.4)	11 614.0 (4.4)	3653.0 (4.5)	−0.15 (−0.31 0.02)
Missing data	222 213 (39.2)	177 705 (40.2)	44 508 (35.6)	
SIMD 10th:				
1st (most deprived)	75 308.0 (13.3)	59 471.0 (13.5)	15 837.0 (12.7)	0.78 (0.57 to 0.99)
2nd	69 135.0 (12.2)	54 502.0 (12.4)	14 633.0 (11.7)	0.62 (0.41 to 0.82)
3rd	63 232.0 (11.2)	49 472.0 (11.2)	13 760.0 (11.0)	0.18 (−0.02 to 0.38)
4th	59 107.0 (10.5)	46 176.0 (10.5)	12 931.0 (10.4)	0.09 (−0.10 to 0.29)
5th	55 315.0 (9.8)	43 799.0 (9.9)	11 516.0 (9.2)	0.69 (0.51 to 0.88)
6th	52 027.0 (9.2)	41 033.0 (9.3)	10 994.0 (8.8)	0.48 (0.30 to 0.66)
7th	51 416.0 (9.1)	40 157.0 (9.1)	11 259.0 (9.0)	0.07 (−0.11 to 0.25)
8th	50 311.0 (8.9)	38 369.0 (8.7)	11 942.0 (9.6)	−0.88 (−1.07 to −0.70)
9th	46 750.0 (8.3)	35 751.0 (8.1)	10 999.0 (8.8)	−0.72 (−0.90 to −0.54)
10th (least deprived)	42 941.0 (7.6)	32 204.0 (7.3)	10 737.0 (8.6)	−1.31 (−1.49 to −1.14)
Missing data	1674 (0.3)	1258 (0.3)	416 (0.3)	
Smoker status:				
Current smoker	102 786.0 (19.0)	82 776.0 (19.6)	20 010.0 (16.8)	2.81 (2.56 to 3.05)
Former smoker	69 282.0 (12.8)	50 644.0 (12.0)	18 638.0 (15.6)	−3.64 (−3.87 to −3.42)
Never smoked, non-smoker	369 988.0 (68.3)	289 346.0 (68.4)	80 642.0 (67.6)	0.84 (0.54 to 1.14)
Missing data	25 160 (4.4)	19 426 (4.3)	5734 (4.6)	
Illicit drug use	3151.0 (0.8)	2528.0 (0.8)	623.0 (0.7)	0.13 (0.07 to 0.20)
Missing data	179 284 (31.6)	141 951 (32.1)	37 333 (29.9)	
Induction of labour	175 239.0 (31.3)	117 798.0 (26.9)	57 441.0 (46.6)	−19.66 (−19.96 to −19.35)
Missing data	6751 (1.2)	4993 (1.1)	1758 (1.4)	
Parity	2.0 (1.0 2.0)	2.0 (1.0 2.0)	1.0 (1.0 2.0)	0.46 (0.45 to 0.46)
Missing data	2885 (0.5)	2290 (0.5)	595 (0.5)	
Multiple birth	6273.0 (1.1)	4026.0 (0.9)	2247.0 (1.8)	−0.89 (−0.97 to −0.81)
No of previous caesarean sections:				
0	534 466.0 (94.9)	417 318.0 (95.1)	117 148.0 (94.2)	0.95 (0.80 to 1.09)
1	28 148.0 (5.0)	20 987.0 (4.8)	7161.0 (5.8)	−0.97 (−1.12 to −0.83)
≥2	352.0 (0.1)	301.0 (0.1)	51.0 (0.0)	0.03 (0.01 to 0.04)
Missing data	4250	3586	664	
Diabetes	13 378.0 (2.4)	9825.0 (2.3)	3553.0 (2.9)	−0.63 (−0.73 to −0.52)
Missing data	20 464 (3.6)	16 507 (3.7)	3957 (3.2)	
Pre-eclampsia status:				
Normal	559 534.0 (98.6)	437 021.0 (98.8)	122 513.0 (98.0)	0.84 (0.75 to 0.92)
Pre-eclampsia	7682.0 (1.4)	5171.0 (1.2)	2511.0 (2.0)	−0.84 (−0.92 to −0.75)
Median (IQR) estimated gestation (weeks)	40.0 (39.0-40.0)	40.0 (38.0-40.0)	40.0 (39.04-1.0)	−0.39 (−0.40 to −0.38)
Preterm	39 601.0 (7.0)	33 564.0 (7.6)	6037.0 (4.8)	2.76 (2.62 to 2.90)
Median (IQR) birthweight (g)	3430.0 (3080.0-3770.0)	3420.0 (3060.0-3760.0)	3490.0 (3147.0-3820.0)	−94 (−98 to −91)
Missing data	924 (0.2)	768 (0.2)	156 (0.1)	
Mode of delivery:				
Spontaneous vaginal delivery	379 009.0 (66.8)	332 829.0 (75.3)	46 180.0 (36.9)	38.33 (38.03 to 38.63)
Breech	1706.0 (0.3)	1593.0 (0.4)	113.0 (0.1)	0.27 (0.25 to 0.29)
Emergency caesarean section	105 288.0 (18.6)	67 075.0 (15.2)	38 213.0 (30.6)	−15.40 (−15.67 to −15.12)
Instrumental	69 645.0 (12.3)	35 206.0 (8.0)	34 439.0 (27.5)	−19.58 (−19.84 to −19.32)
Rotational	11 568.0 (2.0)	5489.0 (1.2)	6079.0 (4.9)	−3.62 (−3.75 to −3.50)
Location:				0.22 (0.21 to 0.23)
Obstetric unit	539 260.0 (95.1)	416 365.0 (94.2)	122 895.0 (98.3)	−5.84 (−5.91 to −5.77)
Freestanding midwifery unit	27 538.0 (4.9)	25 409.0 (5.7)	2129.0 (1.7)	5.75 (5.68 to 5.82)
Home	409.0 (0.1)	409.0 (0.1)	0.0 (0.0)	0.09 (0.08 to 0.10)
Missing data	9 (0.0)	9 (0.0)	0 (0.0)	
Bateman index* (categorical):				
0	456 276.0 (80.4)	353 969.0 (80.0)	102 307.0 (81.8)	−1.78 (−2.03 to −1.54)
1	91 024.0 (16.0)	72 383.0 (16.4)	18 641.0 (14.9)	1.46 (1.23 to 1.69)
2	18 517.0 (3.3)	14 740.0 (3.3)	3777.0 (3.0)	0.31 (0.20 to 0.42)
≥3	1399.0 (0.2)	1100.0 (0.2)	299.0 (0.2)	0.01 (−0.02 to 0.04)

BMI=body mass index; CI=confidence interval; IQR=interquartile range; SIMD=Scottish index of multiple deprivation.

* Weighted obstetric risk prediction tool including 20 conditions plus maternal age.^33^

We also presented results from non-imputed, complete case analyses (n=309 503) and compared these with our main imputed analyses. In accordance with data regulation guidelines, we redacted any outcome or variable with five or fewer values, or any data that could be used to derive these redacted values.

### Patient and public involvement

This study used anonymised data from national registries, focusing on the analysis of existing information without necessitating new direct contact with participants. Despite the inherent limitations of our approach, including the lack of allocated funding for direct patient involvement, we recognised the importance of incorporating public perspectives into our research. While direct involvement in designing the research question, the outcome measures, and study implementation was not feasible, our motivation was strongly influenced by discussions with members of the public and specific concerns highlighted by patients about maternal morbidity rates. These conversations, along with a priority setting exercise by the James Lind Alliance on the impact of epidural analgesia during labour, shaped our research focus.^16^ Although formal patient and public involvement was not integrated into the study’s design, we engaged with the public by inviting a patient to review our manuscript, whose insights contributed to refining our presentation and interpretation of findings.

## Results

### Study population and baseline characteristics

After exclusions, 567 216 women presented in labour in Scotland between 1 January 2007 and 31 December 2019 (table 1, see supplementary eFigure 1), of whom 39 601 (7.0%) delivered prematurely. Epidural analgesia was administered to 125 024 (22.0%) women. Of the 77 439 women with a medical indication for treatment, epidural analgesia was administered to 19 061 (24.6%) (see supplementary eFigure 1). Mothers who received epidural analgesia during labour were more likely to be primiparous, be from a less deprived socioeconomic group, be a former or non-smoker, be undergoing labour induction, give birth in an obstetric unit, and have a multiple birth, ≥1 comorbidities, a higher birthweight baby, and operative delivery (table 1). SMM occurred in 2412 women (0.43%) and was more commonly observed in those with a medical indication for epidural analgesia (819/77 439, 1.06%) and in women delivering preterm (581/39 601, 1.47%) (table 2 and supplementary eTable 4).

**Table 2:** Observed events and adjusted relative risks for all outcomes for whole cohort

	Crude event rate								Adjusted relative risk* (95% CI)	P value
	All pregnancies (n=567 216)			No epidural analgesia (n=442 192)			Epidural analgesia (n=125 024)			
	No	% (95% CI)		No	% (95% CI)		No	% (95% CI)		
No epidural analgesia (reference group)	–	–		–	–		–	–	1.00	
SMM	2412	0.43 (0.41 to 0.44)		1885	0.43 (0.41 to 0.45)		527	0.42 (0.39 to 0.46)	0.65 (0.50 to 0.85)	0.001
SMM+critical care admission	927	0.16 (0.15 to 0.17)		750	0.17 (0.16 to 0.18)		177	0.14 (0.12 to 0.16)	0.46 (0.29 to 0.73)	0.001
Respiratory morbidity	241	0.04 (0.04 to 0.05)		200	0.05 (0.04 to 0.05)		41	0.03 (0.02 to 0.04)	0.42 (0.16 to 1.15)	0.09

CI=confidence interval; SMM=severe maternal morbidity.

* Adjusted for maternal height, weight, ethnicity, Scottish index of multiple deprivation, gestation at birth, comorbidity before labour using Bateman index weighted score (restricted to period of 180 days preconception to day before delivery), parity, induction of labour, previous caesarean (before period used for Bateman index), year of birth, smoking in pregnancy, and type of delivery unit.

### Temporal trends in SMM

The overall incidence of SMM (irrespective of epidural analgesia status) did not change annually during the study period (relative risk per year 1.00 (95% confidence interval (CI) 0.99 to 1.02, P=0.7) (see supplementary eTables 5 and 6).

### Association between epidural analgesia and SMM and related outcomes

Epidural analgesia during labour was associated with a reduction in SMM (adjusted relative risk 0.65, 95% CI 0.50 to 0.85), SMM plus critical care admission (0.46, 0.29 to 0.73), and respiratory morbidity (0.42, 0.16 to 1.15), although the last of these had limited power with wide confidence intervals (table 2).

**Fig 1:**
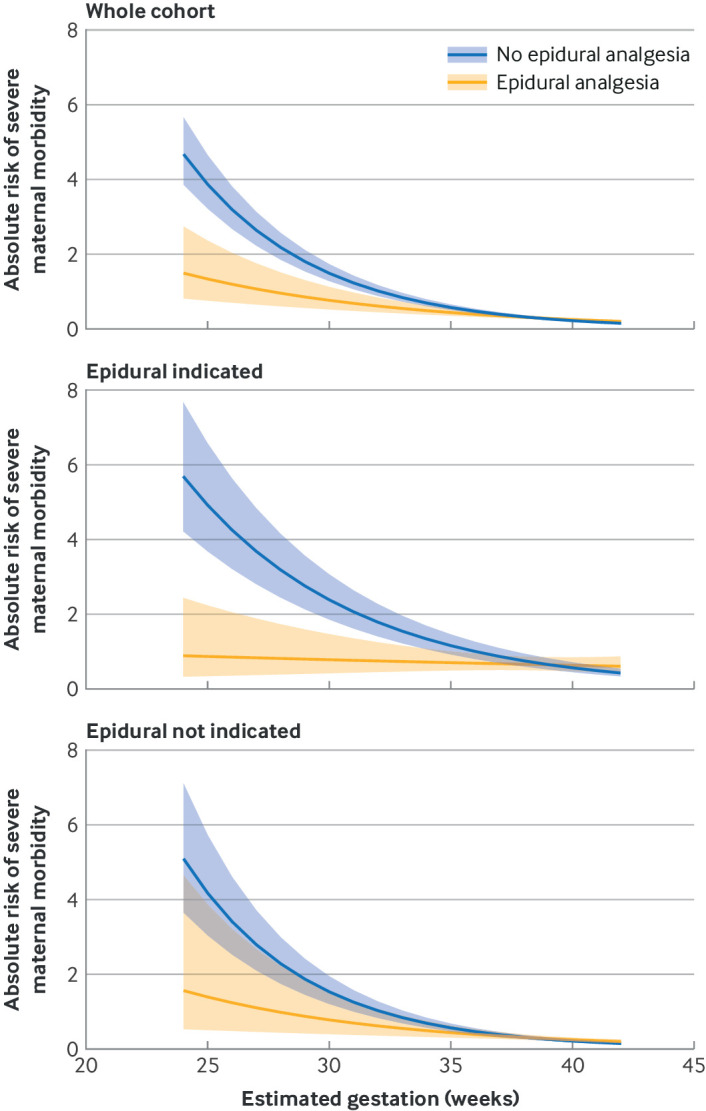
Time varying adjusted absolute risks for severe maternal morbidity (%) in relation to gestational age (in weeks) for whole cohort, women with a medical indication for epidural analgesia, and women with no medical indication for epidural analgesia. Shading represents 95% confidence intervals

In subgroup analyses, epidural analgesia was associated with a greater risk reduction in SMM in women with a medical indication for epidural analgesia (0.50, 0.34 to 0.72) versus those without a medical indication (0.67, 0.43 to 1.03); likelihood ratio of difference between subgroups, P<0.001 (table 3). Similarly, we found a greater risk reduction in SMM in women receiving epidural analgesia and delivering prematurely (0.53, 0.37 to 0.76) compared with women delivering at term or post term (1.09, 0.98 to 1.21); likelihood ratio of difference between subgroups, P<0.001, and in women with a medical indication and delivering prematurely (0.36, 0.24 to 0.53) compared with women with no medical indication and delivering at term or post term (1.14, 0.99 to 1.31); likelihood ratio of difference between subgroups, P<0.001 (table 3). The reduced risk of SMM with epidural analgesia seen in the whole cohort and in women with a medical indication for epidural analgesia was more pronounced as gestational age at birth decreased (fig 1).

**Table 3:** Comparison of outcomes between women with and without a medical indication for epidural analgesia during labour and those delivering preterm compared with at term or post term

	SMM			SMM+critical care admission			Respiratory morbidity	
	Adjusted relative risk (95% CI); P value	P value for difference*		Adjusted relative risk (95% CI); P value	P value for difference*		Adjusted relative risk (95% CI); P value	P value for difference*
Medical indication† (n=77 439)	0.50 (0.34 to 0.72); <0.001	<0.001		0.32 (0.17 to 0.59); <0.001	<0.001		0.51 (0.20 to 1.29); 0.15	<0.001
No medical indication† (n=411 907)	0.67 (0.43 to 1.03); 0.07			0.54 (0.25 to 1.19); 0.13			0.20 (0.04 to 1.12); 0.07	
Preterm birth‡ (n=39 601)	0.53 (0.37 to 0.76); <0.001	<0.001		0.33 (0.17 to 0.63), <0.001	<0.001		0.31 (0.08 to 1.25); 0.10	<0.001
Term/post-term birth‡ (n=527 615)	1.09 (0.98 to 1.21); 0.10			1.05 (0.88 to 1.26); 0.58			0.91 (0.62 to 1.33); 0.62	
Medical indication and preterm birth† (n=12 797)	0.36 (0.24 to 0.53); <0.001	<0.001		0.26 (0.13 to 0.51); <0.001	<0.001		0.49 (0.18 to 1.34); 0.16	<0.001
No medical indication and term/post-term birth† (n=391 813)	1.14 (0.99 to 1.31); 0.06			1.10 (0.86 to 1.39); 0.45			1.14 (0.73 to 1.79); 0.56	

CI=confidence interval; SMM=severe maternal morbidity.

* Derived from likelihood ratio test comparing a model with an interaction between epidural analgesia and the subgroup terms to one without that interaction.

† Adjusted for maternal age, ethnicity, Scottish index of multiple deprivation, gestation at birth, parity, induction of labour, year of birth, smoking in pregnancy, and type of delivery unit.

‡ Adjusted for maternal height, weight, ethnicity, Scottish index of multiple deprivation, gestation at birth, comorbidity before labour using Bateman index weighted score (restricted to period of 180 days preconception to day before delivery), parity, induction of labour, previous caesarean (before period used for Bateman index), year of birth, smoking in pregnancy, and type of delivery unit.

### Robustness of results and sensitivity analysis

E-values suggest our findings are not likely to be solely due to residual confounding (see supplementary eTable 7). Consistent results were observed in analyses limited to births in obstetric units with 24 hour access to obstetric and anaesthetic services (see supplementary eTables 8 and 9). Epidural analgesia was associated with reduced risk of SMM across all categories of preterm birth: extremely preterm (<28 weeks) gestations (0.36, 0.21 to 0.62), very preterm (28 to <32 weeks) gestations (0.48, 0.32 to 0.72), and moderate to late preterm (≥32 to 37 weeks) gestations (0.71, 0.56 to 0.88) (see supplementary eTable 10**)**. This effect was irrespective of whether the reason for the preterm birth was spontaneous or iatrogenic (see supplementary eTable 10). Similar results were seen in both complete case and unimputed datasets (table 2, table 3, and supplementary eTable 11).

## Discussion

In this population based cohort study encompassing 567 216 births in Scotland, epidural analgesia during labour was associated with a 35% risk reduction in SMM and 54% risk reduction in SMM plus critical care admission across all births. These benefits were more pronounced in women with a medical indication for epidural analgesia compared with those without an indication, and in those who delivered preterm compared with those who did not deliver preterm. Women with a higher pre-existing morbidity risk, stemming from either medical or obstetric conditions, spontaneous preterm delivery, or conditions necessitating iatrogenic preterm delivery, face increased risks of adverse events related to their chronic comorbidities, diseases related to preterm birth, haemorrhage, and surgical complications.^4^ ^45^ ^46^ ^47^ Our results suggest that these risks might be effectively mitigated by use of epidural analgesia.

### Comparison with other studies

Our findings enhance the limited existing literature,^8^ ^9^ and respond to a research priority identified by patients and clinical providers.^16^ Given that mode of birth is unknown when the decision to use labour epidural analgesia is made, and that around 15% of SMM events will occur in the postnatal period,^10^ our study provided a more accurate portrayal of the clinical situation than in the previous US study, which did not include postnatal SMM.^8^ As few known modifiable risk factors for SMM exist, and as the incidence of SMM continues to rise, with this increase contributing to the global plateauing of maternal mortality, our findings provide a means to reduce SMM and maternal mortality.^1^ ^4^ ^45^ That a large portion of women in whom epidural analgesia would generally be considered medically indicated did not receive one highlights a potential area for intervention.

The latest UK Mothers and Babies: Reducing Risk through Audits and Confidential Enquiries report underlines the uneven distribution of maternal morbidity and mortality, with deaths in women from black ethnic groups four times higher than in women from white ethnic groups, and the mortality risk twofold higher in women from the most deprived areas compared with least deprived areas.^13^ Recent UK based studies have shown that women from ethnic minority groups and socioeconomically deprived areas are less likely to receive epidural analgesia, although the underlying reasons remain unclear.^14^ ^15^

### Policy implications

Misinformation and misconceptions about epidural analgesia, particularly the effect on delivery mode and neonatal wellbeing, might contribute to inequities in epidural use during labour.^48^ Existing research, including a Cochrane review of 40 randomised controlled trials and two Scottish population based studies, found that epidural analgesia was not causally linked to an increased risk of operative births and did not adversely affect neonatal or long term childhood outcomes, but these studies did not examine SMM or mortality.^11^ ^49^ ^50^ Although a randomised controlled trial would be ideal for confirming our results, the global prevalence of epidural analgesia during labour, its established safety, and the urgency of this research make a strong case for applying our results in clinical practice. Our study offers valuable insights that can potentially reduce inequalities in maternal healthcare by providing robust evidence for individualised, person centred, and informed decision making. To maximise this effect, it is crucial to develop strategies that ensure women from diverse backgrounds, including those in preterm labour, have access to comprehensive information and support about the use of epidural analgesia.

The mechanism by which epidural analgesia could diminish SMM is likely multifaceted, involving closer medical oversight and haemodynamic monitoring, established intravenous access, fluid administration, blunting of physiological stress responses to labour, avoidance of the need for spinal or general anaesthesia for caesarean section, and faster escalation to definitive obstetric interventions. In essence, using epidural analgesia during labour alters the care pathway to one that enhances the capacity to manage adverse events. From these data it is not possible to separate the direct influence of epidural analgesia from the accompanying comprehensive care package. In the UK, implementing epidural analgesia inherently includes this bundle of enhanced care, which could be particularly advantageous for women at heightened risk of SMM.

### Strengths and limitations of this study

Our study was undertaken in a large, unselected population cohort of linked mother-infant data over a 13 year period reflecting contemporary obstetric and anaesthetic practices. We adjusted for confounding variables that were defined before analyses started, used imputation for missing confounder data, and showed consistency between the confounder imputed and complete case analyses. The E-value suggested that bias due to unknown confounders was unlikely to have made a major contribution to our results, and additional sensitivity analyses support the robustness of our findings. We had too few cases of respiratory morbidity to provide precise estimates, highlighting the need for larger studies to explore this outcome. As other forms of anaesthesia may be used in more urgent clinical scenarios, such as major haemorrhage, this could have resulted in more favourable results in the epidural analgesia group. Nevertheless, our analysis aimed to reflect the divergent management pathways and outcomes depending on womens’ choice about epidural analgesia during labour. For instance, a woman with a functioning epidural is potentially more likely to undergo an assisted vaginal delivery than a caesarean section. In line with other UK based studies, we only accounted for postpartum haemorrhage when it necessitated critical care admission, potentially underestimating this morbidity. As a result, our findings might have been attenuated towards the null and strengthens our confidence in the effect seen between epidural analgesia and SMM. Our study excluded elective caesarean births, acknowledging that women undergo this procedure before labour starts and therefore by definition will not receive epidural analgesia during labour. While this analysis was not within our study’s scope, we recognise the importance of investigating anaesthetic choices in elective caesarean deliveries in future research, given the different risk profiles. We used widely validated area deprivation indices to indicate socioeconomic status.^32^ However, we acknowledge that this may not always reflect individual socioeconomic positions (eg, well educated or wealthy women living in an area with a high deprivation score). As the population of Scotland is predominantly white, our results might not be generalisable to more diverse populations; however, the similarity of our results to those of a US study with an ethnically diverse population increases confidence in our findings.^8^ We lacked data on systemic opioid use and maternal haemodynamics, both of which would have been valuable in elucidating the mechanisms by which epidural analgesia during labour could reduce the risk of SMM. Additionally, we did not have information on individual care providers and factors influencing maternal decision making about epidural analgesia. These aspects are crucial for understanding and dealing with potential barriers to the adoption of epidural analgesia during labour.

### Conclusions

Our analysis of 567 216 births in Scotland indicates that epidural analgesia during labour is associated with a 35% risk reduction in SMM in all women. This effect was more pronounced in specific groups, showing a 50% risk reduction in women with predefined risk factors, and a 47% reduction in those delivering prematurely. These findings substantiate the current practice of recommending epidural analgesia during labour to women with known risk factors, underscores the importance of ensuring equitable access to such treatment, and highlights the importance of supporting women from diverse backgrounds to be able to make informed decisions relating to epidural analgesia during labour.

**What is already known on this topic**

Severe maternal morbidity (SMM) is a potentially life threatening outcome of pregnancy
Epidural analgesia during labour may reduce SMM, although evidence is limited
Assessing the effect of epidural analgesia during labour on obstetric outcomes is a research priority for women and healthcare providers

**What this study adds**

This study showed a reduced risk of SMM in women who received epidural analgesia during labour, with the greatest effects seen in those with a medical indication for epidural analgesia or delivering preterm
Encouraging the adoption of, and enhancing accessibility to, epidural analgesia for women in these higher risk categories could be instrumental in improving maternal health outcomes

## Data Availability

Depersonalised study data may be made available on request to accredited researchers who submit a proposal that is approved by NHS Scotland’s electronic Data Research and Innovation Service.

## Web extra

Extra material supplied by authors

Supplementary material: Additional eTables 1-11 and eFigures 1-4

## Notes

Contributors: MS and SNM are joint senior authors. RJK, MS, and SMN conceived and designed the study. All authors acquired, analysed, or interpreted the data. RJK, MS, and SMN drafted the initial manuscript. All authors critically revised the manuscript for important intellectual content. RJK, MS, AK, and LOEH did the statistical analyses. RJK, MS, DAL, and SMN obtained funding. AK and LOEH provided administrative, technical, or material support. MS, DAL, and SMN supervised the study, obtained regulatory approval, and provided advice on analyses. RJK, AK, MS, and SMN had full access to all of the data in the study and take responsibility for the integrity of the data and the accuracy of the data analysis. They are the guarantors. The corresponding author attests that all listed authors meet authorship criteria and that no others meeting the criteria have been omitted.

Transparency: The lead author (RJK) affirms that the manuscript is an honest, accurate, and transparent account of the study being reported, that no important aspects of the study have been omitted, and that any discrepancies from the study as planned (and, if relevant, registered) have been explained.

Dissemination to participants and related patient and public communities: To maximise the impact of our findings, we will employ a multifaceted dissemination strategy, targeting both academic and public audiences. Our plan includes leveraging social media platforms such as X (formerly Twitter) and Facebook to engage with the public, healthcare professionals, and policy makers. We will collaborate with patient advocacy groups (eg, the Maternal Mental Health Alliance (https://maternalmentalhealthalliance.org/) and professional societies such as the Royal College of Obstetricians and Gynaecologists and Obstetric Anaesthetists’ Association to ensure our research reaches a wide audience and is presented in an accessible format, including lay summaries and infographics. Press releases will be distributed to both national and international media outlets, and findings will be presented at national and international conferences to foster academic and clinical discussion. We aim to facilitate a feedback loop by encouraging commentary and discussion through our social media channels, allowing us to gauge public and professional responses to our findings. This feedback will be invaluable for guiding future research directions and policy recommendations, ensuring our work remains aligned with patient needs and priorities.

Provenance and peer review: Not commissioned; externally peer reviewed.
