# Supplementary material for: Epidural analgesia during labour and severe maternal morbidity: population based study
Source: BMJ. 2024 May 22;385:e077190. doi: 10.1136/bmj-2023-077190 (PMC11109902; doi:10.1136/bmj-2023-077190)
Supplement: Supplementary file 1 — Supplementary material: Additional eTables 1-11 and eFigures 1-4 [file kear077190.ww.pdf]

Epidural analgesia in labour and severe maternal morbidity: a population-based study

Kearns RJ,[1,2] Kyzayeva A,[2] Halliday LOE,[2] Lawlor DA,[3,4] Shaw M,[2,5*] Nelson SM[2*]

Dr Rachel J Kearns (https://orcid.org/0000-0001-6156-6858), consultant anaesthetist (rachel.kearns@glasgow.ac.uk)[1,2]

Dr Aizhan Kyzayeva, research associate (aizhan.kyzayeva@glasgow.ac.uk)[2]

Dr Lucy OE Halliday, doctoral student (lucy.halliday2@nhs.scot)[2]

Professor Deborah A Lawlor, professor of epidemiology. (d.a.lawlor@bristol.ac.uk)[3,4]

Dr Martin Shaw, principle clinical physicist (martin.shaw@ggc.scot.nhs.uk)[2,5*]

Professor Scott Nelson, Muirhead chair of reproductive and maternal medicine, University of Glasgow (scott.nelson@glasgow.ac.uk)[2*]

Affiliations

1. Department of anaesthesia, Glasgow Royal Infirmary, UK
2. School of medicine, University of Glasgow, UK
3. MRC Integrative Epidemiology Unit at the University of Bristol, UK
4. Population Health Science, University of Bristol, Bristol, UK
5. Department of Medical Physics and Bioengineering, NHS Greater Glasgow and Clyde, UK

* Denotes joint last authors

Corresponding author:

Dr Rachel J Kearns, School of Medicine, University of Glasgow, 2nd Floor, Lister Building, Glasgow Royal Infirmary, 10-16, Alexandra Parade, Glasgow, G31 2ER, United Kingdom.

ORCID ID: https://orcid.org/0000-0001-6156-6858

Email: rachel.kearns@glasgow.ac.uk Tel: 0141 9560532 X: @rjharrison79

**eTable 1**	**US Centers for Disease Control and Prevention criteria with ICD-9, ICD-10 and OPCS codes used to components of Severe Maternal Morbidity and respiratory complications diagnosed from date of delivery to 42 days postnatally, with modifications made to these in this study.**
**eTable 2**	**Bateman Index: List of conditions and associated weights. All conditions were diagnosed within the time period of 180-days prior to the estimated date of conception and the day before delivery.**
**eTable 3**	**ICD-9 and ICD-10 codes used to define "medical indication for epidural" and contraindications for epidural analgesia. All conditions diagnosed prior to date of delivery.**
**eTable 4**	**Observed events (n, %), and adjusted relative risks (RR) and 95% CI for all outcomes comparing labour epidural to no epidural in all participants by subgroups.**
**eTable 5**	**Observed events for all components of SMM outcome variable (unimputed).**
**eTable 6**	**Change in each SMM component per year over the study period of 1st January 2007 and 31st December 2019 (unimputed).**
**eTable 7**	**Sensitivity analysis to explore unmeasured confounding using E-value for point estimate.**
**eTable 8**	**Observed events (n, %), and adjusted relative risks (RR) and 95% CI for all outcomes for cohort restricted to obstetric units (N=541,389).**
**eTable 9**	**Comparison of adjusted relative risks (RR) and 95% CI for all outcomes comparing labour epidural to no epidural in; preterm and term/post-term births, and in women with and without a medical indication for epidural for cohort restricted to obstetric units (N=541,389).**
**eTable 10**	**Adjusted relative risks (RR) and 95% CI for all outcomes referent to receiving no epidural analgesia (RR = 1) by WHO category of preterm birth: "extremely preterm" (< 28 weeks), "very preterm" (28 to < 32 weeks), and "moderate to late preterm" (≥ 32 to 36+6 weeks), and by category of spontaneous or iatrogenic preterm birth.**
**eTable 11**	**Observed events (n, %), unadjusted and adjusted relative risks (RR) and 95% CI for all outcomes for whole cohort on unimputed data set.**

**eFigure 1**	**Definition of cohort for analysis**
**eFigure 2**	**Incidence of sepsis compared with incidence of sepsis plus critical care admission over time.**
**eFigure 3a**	**Directed Acyclic Graph (DAG) for analysis of whole cohort and preterm births.**
**eFigure 3b**	**Directed Acyclic Graph (DAG) for analyses stratified by medical indication for epidural**
**eFigure 4**	**Adjusted relative risk for all components of SMM compared with not having an epidural (RR=1)**

eTable 1 **US Centers for Disease Control and Prevention criteria with ICD-9, ICD-10, and OPCS codes* used to define components of SMM and respiratory complications diagnosed from date of delivery to 42 days postnatally, with modifications made to these in this study**

SMM Indicator	ICD-9	ICD-10	Criteria
**SMM**			
Acute myocardial infarction	410.xx	I21, I22	CDC SMM criteria (no modifications).
Aneurysm	441.xx	I71, I79	CDC SMM criteria (no modifications).
Acute renal failure	584.5, 584.6, 584.7, 584.8, 584.9, 669.3x	N17, O90.4	CDC SMM criteria (no modifications).
Adult respiratory distress syndrome	518.5x, 518.81 518.82 518.84, 799.1	J80, J95.1, J95.2, J95.3, J95.8, J96.0, J96.2, R09.2	CDC SMM criteria (no modifications).
Amniotic fluid embolism	673.1x	O88.1x	CDC SMM criteria (no modifications).
Cardiac arrest/ventricular fibrillation	427.41, 427.42*, 427.5	I46.x, I49.0x	CDC SMM criteria (no modifications).
Conversion of cardiac rhythm		X50[124]	CDC SMM criteria (no modifications).
Disseminated intravascular coagulation	286.6, 286.9, 666.3x	D65, D68.8, D68.9, O72.3	CDC SMM criteria (no modifications).
Eclampsia	642.6x	O15.xx	CDC SMM criteria (no modifications).
Heart failure/arrest during surgery or procedure	997.1	I97.12x, I97.13x, I97.710, I97.711	CDC SMM criteria (no modifications).
Puerperal cerebrovascular disorders	430.xx, 431.xx, 432.xx, 433.xx, 434.xx, 436xx, 437.xx, 671.5x, 674.0x, 997.02	I60.xx, I61.xx, I62.xx, I63.xx, I64.xx, I65.xx, I66.xx, I67.xx, I68.xx, O22.51, O22.52, O22.53, I97.81x, I97.82x, O87.3	CDC SMM criteria (no modifications).
Pulmonary edema / Acute heart failure	518.4, 428.1, 428.0, 428.21, 428.23, 428.31, 428.33, 428.41, 428.43	J81.0, I50.1, I50.20, I50.21, I50.23, I50.30, I50.31, I50.33, I50.40, I50.41, I50.43, I50.9	CDC SMM criteria (no modifications).

Severe anesthesia complications	668.0x, 668.1x, 668.2x	O74.0, O74.1, O74.2, O74.3, O74.9, O89.0x, O89.1, O89.2	CDC SMM criteria (no modifications).
Sepsis	038.xx, 995.91, 995.92, 670.2x	O85, O86.04, T80.211A, T81.4XXA, T81.44, R65.20, A40.0x, A41.x, A32.7	CDC SMM criteria plus additional criteria of admission to critical care unit as identified from SICSAG dataset.
Shock	669.1x, 785.5x, 995.0, 995.4, 998.0x	O75.1, R57.0, R57.1, R57.8, R57.9, R65.21, T78.2, T81.10, T81.11, T81.19	CDC SMM criteria (no modifications).
Sickle cell disease with crisis	282.42, 282.62, 282.64, 282.69	D57.0x, D57.21x, D57.41x, D57.81x	CDC SMM criteria (no modifications).
Air and thrombotic embolism	415.1x, 673.0x, 673.2x 673.3x, 673.8x	I26.xx, O88.0x, O88.2x, O88.3x, O88.8x	CDC SMM criteria (no modifications).
Hysterectomy	691[246], 744.xx, 764.xx	R251.xx, Q07[123458], Q08	CDC SMM criteria (no modifications).
Temporary tracheostomy	24[78]	E42[12389]	CDC SMM criteria (no modifications).
Ventilation	-	E85[12]	CDC SMM criteria (no modifications).
Postpartum haemorrhage (plus critical care admission as identified from SICSAG dataset)	666[012]	O72	CDC SMM criteria plus additional criteria of admission to critical care unit as identified from SICSAG dataset.
**Respiratory complications**			
Complications of anesthesia during labour and delivery, ventilation, tracheostomy, or ARDS	668.0, 24[78], 518.5x, 518.81 518.82 518.84, 799.1	O74.0, O74,1, O74.7, O89.0, O89.6, J80, J95.1, J95.2, J95.3, J95.8, J96.0, J96.2, R09.2 E85[12],* E42[12389],*	CDC SMM criteria

ARDS=acute respiratory distress syndrome; CDC=US Centers for Disease Control and Prevention; ICD-9=International Classification of Diseases, ninth revision. OPCS=Office of Population Censuses and Surveys Classification of Interventions and Procedures. SICSAG=Scottish Intensive Care Society Audit Group; SMM=severe maternal morbidity

eTable 2 **Bateman Index: List of conditions and associated weights. All conditions were diagnosed within the time period of 180-days prior to the estimated date of conception and the day before delivery.**

Condition	Weighting	ICD-9 code	ICD-10 code
Severe preeclampsia/eclampsia	5	642.5, 646.6	O14, O15
Chronic congestive heart failure	5	428.22, 428.23, 428.32, 428.33, 428.42, 428.43	I50.0
Congenital heart disease	4	745.0-747.4, 648.5	Q20-Q26, O99.4
Pulmonary hypertension	4	416.0, 416.8, 416.9	I27.0, I27.2, I27.8, I27.9
Chronic ischemic heart disease	3	412-414	I20, I25
Sickle cell disease	3	282.4, 282.6	D56, D57
Multiple gestation	2	651, V27.2-V27.8	O30, O31, Z37.2-Z37.7, Z37.90
Cardiac valvular disease	2	394-397, 424	I05-I09, I34-I39
Systemic lupus erythematosus	2	710.0	M32
Human immunodeficiency virus	2	042, V08	B20, B24, O98.7, Z21
Mild or unspecified preeclampsia*	2	642.4, 642.7 (without severe preeclampsia/eclampsia)	O11, O14 (without severe pre-eclampsia/eclampsia)
Drug abuse	2	304, 305.2-305.9, 648.3	F11-F16, F18, F19
Placenta previa	2	641.0, 641.1	O44
Chronic renal disease	1	581-583, 585, 587, 588, 646.2	N02.2, N03-N05, N08, N17.1, N17.2, N18, N25, O26.8
Pre-existing hypertension	1	401-405, 642.0-642.2, 642.7	I10-I13, I15, O10, O11
Previous caesarean delivery	1	654.2	O82
Gestational hypertension	1	642.3 (without preeclampsia/eclampsia or preexisting hypertension)	O11, O14 (without severe pre-eclampsia/eclampsia)
Alcohol abuse	1	291, 303, 305.0	F10
Asthma	1	493	J44, J45
Pre-existing diabetes mellitus	1	250, 648.0	E10, E11, O24.5-O24.7
Maternal age, years			
>44	3	From SMR02	From SMR02

40–44	2	From SMR02	From SMR02
35–39	1	From SMR02	From SMR02

ICD = International Classification of Disease. SMR02 = Scottish Morbidity Record 02.

eTable 3 **ICD-9 and ICD-10 codes used to define "medical indication for epidural" and contraindications for epidural analgesia. All conditions were diagnosed prior to date of delivery.**

**Medical indication for epidural**	**ICD-9 code**	**ICD-10 code**
Pre-eclampsia	642[4567]	O11, O14, O15
Asthma	493	J44, J45
Cardiac valve disease	394, 395, 396, 397, 424	I05-I09, I34-39
Congestive cardiac failure	428.2, 428.3, 428.4	I50
Congenital heart disease	648.5, 745-747	Q20-Q26
Ischaemic heart disease	412-414	I20, I25
Pulmonary hypertension	416.0, 416.8, 416.9	I27.0, I27.2, I27.8, I27.9
Previous caesarean section	654.2	O82
Multiple gestation	651, V27.2-V27.8	O30, O31, Z37.2-Z37.7, Z37.90
Breech presentation	From SMR02	From SMR02
BMI $\geq$ 40	From SMR02	From SMR02
**Contraindications**		
Coagulation factor deficit, Von Willebrand disease, and thrombocytopenia	286, 287	D65-D69
Fever or infection during labour	659.2, 659.3	O75.2, O75.3
Chorioamnionitis	658.4	O41.1

ICD = International Classification of Disease, SMR02 = Scottish Morbidity Record 02.

eTable 4 **Observed events (n, %), and adjusted relative risks (RR) and 95% CI for all outcomes comparing labour epidural to no epidural in all participants by subgroups.**

	**Crude Event Rate N (%), [95% CI]**			**Adjusted RR (95% CI); P value**
**Medical Indication† (N=77,439)**				
**Outcome**	**All pregnancies within the subgroup**	**No epidural**	**Epidural**	
SMM	819/77,439 (1.06%) [0.99%, to1.1%]	645/58,378 (1.10%) [1.0% to 1.2%]	174/19,061 (0.91%) [0.78% to 1.1%]	0.50 (0.34 to 0.72); <0.001
SMM plus critical care admission	331/77,439 (0.43%) [0.38% to 0.48%]	277/58,378 (0.47%) [0.42% to 0.53%]	54/19,061 (0.28%) [0.21% to 0.37%]	0.32 (0.17 to 0.59); 0.003
Respiratory morbidity	82/77,439 (0.11%) [0.08% to 0.13%]	73/58,378 (0.13%) [0.10% to 0.16%]	9/19,061 (0.05%) [0.02% to 0.09%]	0.51 (0.199 to 1.29); 0.15
**No Medical Indication† (N=411,907)**				
**Outcome**	**All pregnancies**	**No epidural**	**Epidural**	
SMM	1,193/411,907 (0.29%) [0.27% to 0.31%]	915/321,945 (0.28%) [0.27% to 0.30%]	278/89,962 (0.31%) [0.27% to 0.35%]	0.67 (0.43 to 1.03); 0.07
SMM plus critical care admission	455/411,907 (0.11%) [0.10% to 0.12%]	359/321,945 (0.11%) [0.10% to 0.12%]	96/89,962 (0.11%) [0.09% to 0.13%]	0.54 (0.25 to 1.19); 0.13
Respiratory morbidity	120/411,907 (0.03%) [0.02% to 0.03%]	95/321,945 (0.03%) [0.02% to 0.04%]	25/89,962 (0.03%) [0.02% to 0.04%]	0.20 (0.04 to 1.12); 0.07
**Preterm birth‡ (N=39,601)**				
**Outcome**	**All pregnancies**	**No epidural**	**Epidural**	
SMM	581/39,601 (1.47%) [1.4% to 1.6%]	530/33,564 (1.58%) [1.4% to 1.7%]	51/6037 (0.84%) [0.64% to 1.1%]	0.53 (0.37 to 0.76); <0.001
SMM plus critical care admission	297/39,601 (0.75%) [0.67% to 0.84%]	277/33,564 (0.83%) [0.73% to 0.93%]	20/6037 (0.33%) [0.21% to 0.52%]	0.33 (0.17 to 0.63), <0.001

Respiratory morbidity	75/39,601 (0.19%) [0.15% to 0.24%]	*/33,564 (*%) [0.16% to 0.27%]	*/6,037 (*%) [0.03% to 0.21%]	0.31 (0.08 to 1.25); 0.10
**Term / post-term birth‡ (N=527,615)**				
**Outcome**	**All pregnancies**	**No epidural**	**Epidural**	
SMM	1,831/527,615(0.35%), [0.33% to 0.36%]	1,355/408,628 (0.33%), [0.31% to 0.35%]	476/118,987 (0.40%), [0.37% to 0.44%]	1.09 (0.98 to 1.21); 0.10
SMM plus critical care admission	630/527,615 (0.12%), [0.11% too 0.13%]	473/408,628 (0.12%), [0.11% to 0.13%]	157/118,987 (0.13%), [0.11% to 0.15%]	1.05 (0.88 to 1.26); 0.58
Respiratory morbidity	166/527,615 (0.03%), [0.03% to 0.04%]	130/408,628 (0.03%), [0.03% to 0.04%]	36/118,987 (0.03%), [0.02% to 0.04%]	0.91 (0.62 to 1.33); 0.62
**Medical Indication and preterm birth† (N=12,797)**				
**Outcome**	**All pregnancies**	**No epidural**	**Epidural**	
SMM	287/12,797 (2.24%) [2.0% to 2.5%]	267/10,546 (2.53%) [2.2% to 2.9%]	20/2,251 (0.89%) [0.56% to 1.4%]	0.36 (0.24 to 0.53); <0.001
SMM plus critical care admission	149/12,797 (1.16%) [0.99% to 1.4%]	*/10,546 (*%) [1.1% to 1.6%]	*/2,251 (*%) [011% to 0.61%]	0.26 (0.13 to 0.51); <0.001
Respiratory morbidity	38/12,797 (0.30%) [0.21% to 0.41%]	*/10,546 (*%) [0.23% to 0.47%]	*/2,251 (*%) [0.03% to 0.42%]	0.49 (0.18 to 1.34); 0.16
**No medical Indication and term / post-term birth† (N=391,813)**				
**Outcome**	**All pregnancies**	**No epidural**	**Epidural**	
SMM	1,004/391,813 (0.26%) [0.24% to 0.27%]	747/304,755 (0.25%) [0.23% to 0.26%]	257/87,058 (0.30%) [0.26% to 0.33%]	1.14 (0.99 to 1.31); 0.06
SMM plus critical care admission	356/391,813 (0.09%) [0.08% to 0.10%]	270/304,755 (0.09%) [0.08% to 0.10%]	86/87,058 (0.10%) [0.08% to 0.12%]	1.10 (0.87 to 1.39); 0.45
Respiratory morbidity	99/391,813 (0.03%) [0.02% to 0.03%]	74/304,755 (0.02%) [0.02% to 0.03%]	25/87,058 (0.03%) [0.02% to 0.04%]	1.14 (0.73 to 1.79); 0.56

†Adjusted for maternal age, ethnicity, Scottish index of multiple deprivation, gestation at birth, parity, induction of labour, year of birth, smoking in pregnancy, and type of delivery unit.
‡Adjusted for maternal height, weight, ethnicity, Scottish index of multiple deprivation, gestation at birth, comorbidity before labour using Bateman index weighted score (restricted to period of 180 days preconception to day before delivery), parity, induction of labour, previous caesarean (before period used for Bateman index), year of birth, smoking in pregnancy, and type of delivery unit.

*We redacted any outcome or variable with five or fewer value, and any data which could be used to derive these redacted values.

eTable 5 Observed events for all components of SMM (unimputed data).

Characteristic	Overall, N = 567,216[1]	95% CI[2]	No epidural, N = 442,192[1]	Epidural, N = 125,024[1]	Difference[3]	p-value[3]
Amniotic fluid embolism	11 (0.00%)	0.00% to 0.00%	*	*	0.00%	>0.9
Acute heart failure	56 (0.01%)	0.01% to 0.01%	45 (0.01%)	11 (0.01%)	0.00%	>0.9
Severe anesthesia complications	74 (0.01%)	0.01% to 0.02%	63 (0.01%)	11 (0.01%)	0.01%	0.3
Aneurysm	*	0.00% to 0.00%	*	*	0.00%	0.6
Adult respiratory distress syndrome	35 (0.01%)	0.00% to 0.01%	26 (0.01%)	9 (0.01%)	0.00%	0.9
Acute renal failure	174 (0.03%)	0.03% to 0.04%	118 (0.03%)	56 (0.04%)	-0.02%	0.005
Cardiac arrest	20 (0.00%)	0.00% to 0.01%	*	*	0.00%	0.2
Puerperal cerebrovascular disorders	70 (0.01%)	0.01% to 0.02%	51 (0.01%)	19 (0.02%)	0.00%	0.6
Conversion of cardiac rhythm	*	0.00% to 0.00%	*	*	0.00%	0.4
Disseminated Intravascular Coagulation	416 (0.07%)	0.07% to 0.08%	342 (0.08%)	74 (0.06%)	0.02%	0.11
Eclampsia	265 (0.05%)	0.04% to 0.05%	194 (0.04%)	71 (0.06%)	-0.01%	0.2
Heart failure/arrest during surgery	0 (0.00%)	0.00% to 0.00%	0 (0.00%)	0 (0.00%)	0.00%	
Hysterectomy	23 (0.00%)	0.00% to 0.01%	*	*	0.00%	0.6
Acute myocardial infarction	13 (0.00%)	0.00% to 0.00%	*	*	0.00%	0.4

Characteristic	Overall, N = 567,216[1]	95% CI[2]	No epidural, N = 442,192[1]	Epidural, N = 125,024[1]	Difference[3]	p-value[3]
Postpartum hemorrhage associated with critical care admission	799 (0.14%)	0.13% to 0.15%	642 (0.15%)	157 (0.13%)	0.02%	0.3
Sepsis	1,693 (0.30%)	0.28% to 0.31%	860 (0.19%)	833 (0.67%)	-0.47%	<0.001
Sepsis associated with critical care admission	119 (0.02%)	0.02% to 0.03%	93 (0.02%)	26 (0.02%)	0.00%	>0.9
Shock	134 (0.02%)	0.02% to 0.03%	99 (0.02%)	35 (0.03%)	-0.01%	0.5
Sickle cell disease with crisis	8 (0.00%)	0.00% to 0.00%	*	*	0.00%	0.2
Tracheostomy	6 (0.00%)	0.00% to 0.00%	6 (0.00%)	0 (0.00%)	0.00%	0.4
Ventilation	138 (0.02%)	0.02% to 0.03%	123 (0.03%)	15 (0.01%)	0.02%	0.007
Air and thrombotic embolism	282 (0.05%)	0.04% to 0.06%	228 (0.05%)	54 (0.04%)	0.01%	0.5

NB – an individual patient may have had ≥1 SMM event. CI = 95% Confidence Interval
[1]n (%), [2]Fisher's exact test; [3]Pearson's Chi-squared test,
*We redacted any outcome or variable with five or fewer values, or any data which could be used to derive these redacted values.

eTable 6 **Change in each SMM component per year over the study period of 1st January 2007 and 31st December 2019 (unimputed data).**

Group	RR	95% CI	p-value
Ventilation	0.99	0.95 to 1.02	0.5
Tracheostomy	0.94	0.78 to 1.12	0.5
Hysterectomy	0.91	0.78 to 1.06	0.2
Conversion of cardiac rhythm	0.96	0.74 to 1.23	0.7
Acute myocardial infarction	0.98	0.84 to 1.13	0.7
Aneurysm	1.23	0.97 to 1.68	0.12
Acute renal failure	1.19	1.13 to 1.26	<0.001
Adult respiratory distress syndrome	0.97	0.91 to 1.04	0.5
Amniotic fluid embolism	1.14	1.00 to 1.32	0.07
Cardiac arrest	1.04	0.92 to 1.18	0.5
Disseminated intravascular coagulation	0.96	0.93 to 0.99	0.02
Eclampsia	0.94	0.89 to 1.01	0.07
Heart failure/arrest during surgery	1.00	0.84 to 1.19	>0.9
Puerperal cerebrovascular disorders	0.99	0.94 to 1.04	0.6
Acute Heart failure	0.94	0.89 to 0.99	0.02
Severe anesthesia complications	0.81	0.74 to 0.89	<0.001
Sepsis	1.29	1.25 to 1.34	<0.001
Postpartum hemorrhage	1.06	1.05 to 1.07	<0.001
Shock	1.01	0.96 to 1.06	0.8
Sickle cell disease with crisis	0.89	0.73 to 1.06	0.2

Group	RR	95% CI	p-value
Air and thrombotic embolism	1.05	1.01 to 1.09	0.006
Sepsis associated with critical care admission	1.05	1.01 to 1.09	0.01
Postpartum hemorrhage associated with critical care admission	1.03	1.01 to 1.05	0.02
Severe maternal morbidity (total)	1.00	0.99 to 1.02	0.7

RR = Relative Risk. 95% CI = 95% Confidence Interval

eTable 7 Sensitivity analysis to explore unmeasured confounding using E-value for point estimate.

**Cohort**	**Characteristic**	**e-value for RR**
Whole cohort	SMM	2.44
	SMM plus critical care admission	3.77
	Respiratory morbidity	4.19
Medical indication for epidural	SMM	3.45
	SMM plus critical care admission	5.72
	Respiratory morbidity	3.37
No medical indication for epidural	SMM	2.35
	SMM plus critical care admission	3.10
	Respiratory morbidity	9.52
Preterm	SMM	3.18
	SMM plus critical care admission	5.51
	Respiratory morbidity	5.91
Term / post-term	SMM	1.40
	SMM plus critical care admission	1.28
	Respiratory morbidity	1.43

SMM = Severe Maternal Morbidity, RR = Relative Risk

eTable 8 Observed events (n, %), and adjusted relative risks (RR) and 95% CI for all outcomes for cohort restricted to obstetric units (N=541,389).

	Crude event rate							
	All pregnancies (n=541,389)		No epidural analgesia (n=416,365)		Epidural analgesia (n=125 024)		Adjusted relative risk* (95% CI)	P value
	No	% (95% CI)	No	% (95% CI)	No	% (95% CI)		
No epidural analgesia (reference group)	–	–	–	–	–	–	1.00	
SMM	2315	0.43 (0.41 to 0.45)	1788	0.43 (0.41 to 0.45)	527	0.42 (0.39 to 0.46)	0.65 (0.50 to 0.85)	0.001
SMM+critical care admission	900	0.17 (0.16 to 0.18)	723	0.17 (0.16 to 0.19)	177	0.14 (0.12 to 0.16)	0.46 (0.29 to 0.74)	0.001
Respiratory morbidity	235	0.04 (0.04 to 0.05)	194	0.05 (0.04 to 0.05)	41	0.03 (0.02 to 0.04)	0.43 (0.16 to 1.16)	0.10

CI=confidence interval; SMM=Severe Maternal Morbidity.
*Adjusted for maternal height, weight, ethnicity, Scottish index of multiple deprivation, gestation at birth, comorbidity before labour using Bateman index weighted score (restricted to period of 180 days preconception to day before delivery), parity, induction of labour, previous caesarean (before period used for Bateman index), year of birth, and smoking in pregnancy.

eTable 9 **Comparison of adjusted relative risks (RR) and 95% CI for all outcomes comparing labour epidural to no epidural in; preterm and term/post-term births, and in women with and without a medical indication for epidural for cohort restricted to obstetric units (N=541,389).**

	**SMM**			**SMM+critical care admission**			**Respiratory morbidity**	
	**Adjusted relative risk (95% CI); P value**	**P value for difference***		**Adjusted relative risk (95% CI); P value**	**P value for difference***		**Adjusted relative risk (95% CI); P value**	**P value for difference***
Medical indication† (n=76 006)	0.50 (0.35 to 0.73); <0.001	<0.001		0.33 (0.18 to 0.60); <0.001	<0.001		0.51 (0.20 to 1.29); 0.15	<0.001
No medical indication† (n=394 676)	0.67 (0.43 to 1.03); 0.07			0.54 (0.24 to 1.19); 0.12			0.20 (0.04 to 1.12); 0.07	
Preterm birth‡ (n=38 792)	0.55 (0.35 to 0.85); 0.007	<0.001		0.33 (0.17 to 0.64); 0.001	<0.001		0.31 (0.08 to 1.25); 0.10	<0.001
Term/post-term birth‡ (n=502 597)	1.13 (1.02 to 1.26); 0.02			1.04 (0.87,1.25); 0.65			0.91 (0.62 to 1.33); 0.62	
Medical indication and preterm birth† (n=12 679)	0.36 (0.24 to 0.54); <0.001	<0.001		0.27 (0.14 to 0.52); <0.001	<0.001		0.49 (0.18 to 1.34); 0.16	<0.001
No medical indication and term/post-term birth† (n=374 869)	1.14 (0.99 to 1.31); 0.06			1.07 (0.85 to 1.36); 0.55			1.14 (0.73 to 1.79); 0.56	

*Derived from likelihood ratio test comparing a model with an interaction between epidural analgesia and the subgroup terms to one without that interaction.
†Adjusted for maternal age, ethnicity, Scottish index of multiple deprivation, gestation at birth, parity, induction of labour, year of birth, and smoking in pregnancy.
‡Adjusted for maternal height, weight, ethnicity, Scottish index of multiple deprivation, gestation at birth, comorbidity before labour using Bateman index weighted score (restricted to period of 180 days preconception to day before delivery), parity, induction of labour, previous caesarean (before period used for Bateman index), year of birth, smoking in pregnancy.

eTable 10 **Adjusted relative risks (RR) and 95% CI for all outcomes referent to receiving no epidural analgesia (RR = 1) by WHO category of preterm birth: “extremely preterm” (< 28 weeks), “very preterm” (28 to < 32 weeks), and “moderate to late preterm” (≥ 32 to 36+6 weeks), and by category of spontaneous or iatrogenic preterm birth.**

Group	Characteristic	RR	95% CI[1]	p-value
Moderate preterm‡	SMM	0.71	0.56 to 0.88	0.002
	SMM plus critical care admission	0.52	0.35 to 0.78	0.002
	Respiratory morbidity	0.48	0.20 to 1.12	0.09
Very preterm‡	SMM	0.48	0.32 to 0.72	<0.001
	SMM plus critical care admission	0.28	0.13 to 0.58	<0.001
	Respiratory morbidity	0.27	0.05 to 1.31	0.10
Extreme preterm‡	SMM	0.36	0.21 to 0.62	<0.001
	SMM plus critical care admission	0.18	0.07 to 0.48	<0.001
	Respiratory morbidity	0.18	0.02 to 1.50	0.11
Spontaneous preterm‡	SMM	0.55	0.38 to 0.78	0.001
	SMM plus critical care admission	0.42	0.24 to 0.73	0.002
	Respiratory morbidity	0.36	0.11 to 1.15	0.09
Iatrogenic preterm‡	SMM	0.54	0.32 to 0.93	0.03
	SMM plus critical care admission	0.53	0.24 to 1.20	0.13
	Respiratory morbidity	0.84	0.20 to 3.61	0.80

‡ Adjusted for maternal height, weight, ethnicity, SIMD, gestation at birth, co-morbidity prior to labour using Bateman index weighted score (restricted to period of 180 days pre-conception to day before delivery), parity, induction of labour, previous caesarean (prior to the time period used for the Bateman index), year of birth, smoking in pregnancy, type of delivery unit.
SIMD = Scottish Index of Multiple Deprivation, SMM = Severe Maternal Morbidity, RR = Relative Risk, CI = Confidence Interval

eTable 11 Observed events (n, %), unadjusted and adjusted relative risks (RR) and 95% CI for all outcomes for whole cohort on unimputed complete case data set.

	Crude event rate									
	All pregnancies (n=567 216)		No epidural analgesia (n=442 192)		Epidural analgesia (n=125 024)		Unadjusted relative risk (95% CI)	P value	Adjusted relative risk* (95% CI)	P value
	No	% (95% CI)	No	% (95% CI)	No	% (95% CI)				
No epidural analgesia (reference group)	–	–	–	–	–	–	1.00	–	1.00	–
SMM	2412	0.43 (0.41 to 0.44)	1885	0.43 (0.41 to 0.45)	527	0.42 (0.39 to 0.46)	0.72 (0.55 to 0.93)	0.01	0.55 (0.38 to 0.80)	0.002
SMM+critical care admission	927	0.16 (0.15 to 0.17)	750	0.17 (0.16 to 0.18)	177	0.14 (0.12 to 0.16)	0.49 (0.31 to 0.77)	0.002	0.37 (0.19 to 0.70)	0.003
Respiratory morbidity	241	0.04 (0.04 to 0.05)	200	0.05 (0.04 to 0.05)	41	0.03 (0.02 to 0.04)	0.44 (0.18 to 1.1)	0.08	0.17 (0.04 to 0.75)	0.002

CI=confidence interval; SMM=Severe Maternal Morbidity.
*Adjusted for maternal height, weight, ethnicity, Scottish index of multiple deprivation, gestation at birth, comorbidity before labour using Bateman index weighted score (restricted to period of 180 days preconception to day before delivery), parity, induction of labour, previous caesarean (before period used for Bateman index), year of birth, smoking in pregnancy, and type of delivery unit.

eFigure 1 **Definition of cohort for analysis**

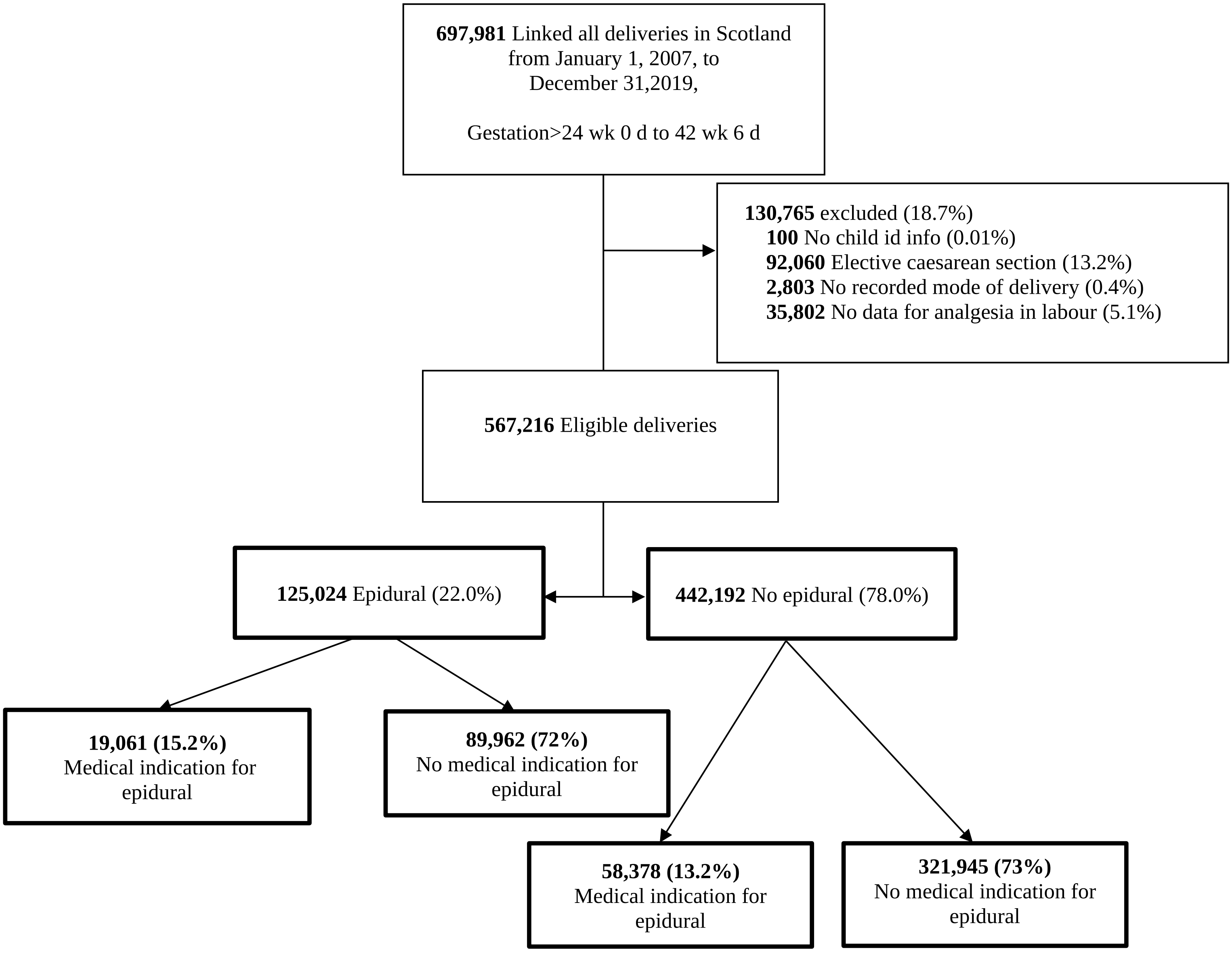

eFigure 2 **Incidence of sepsis compared with incidence of sepsis plus critical care admission over time**

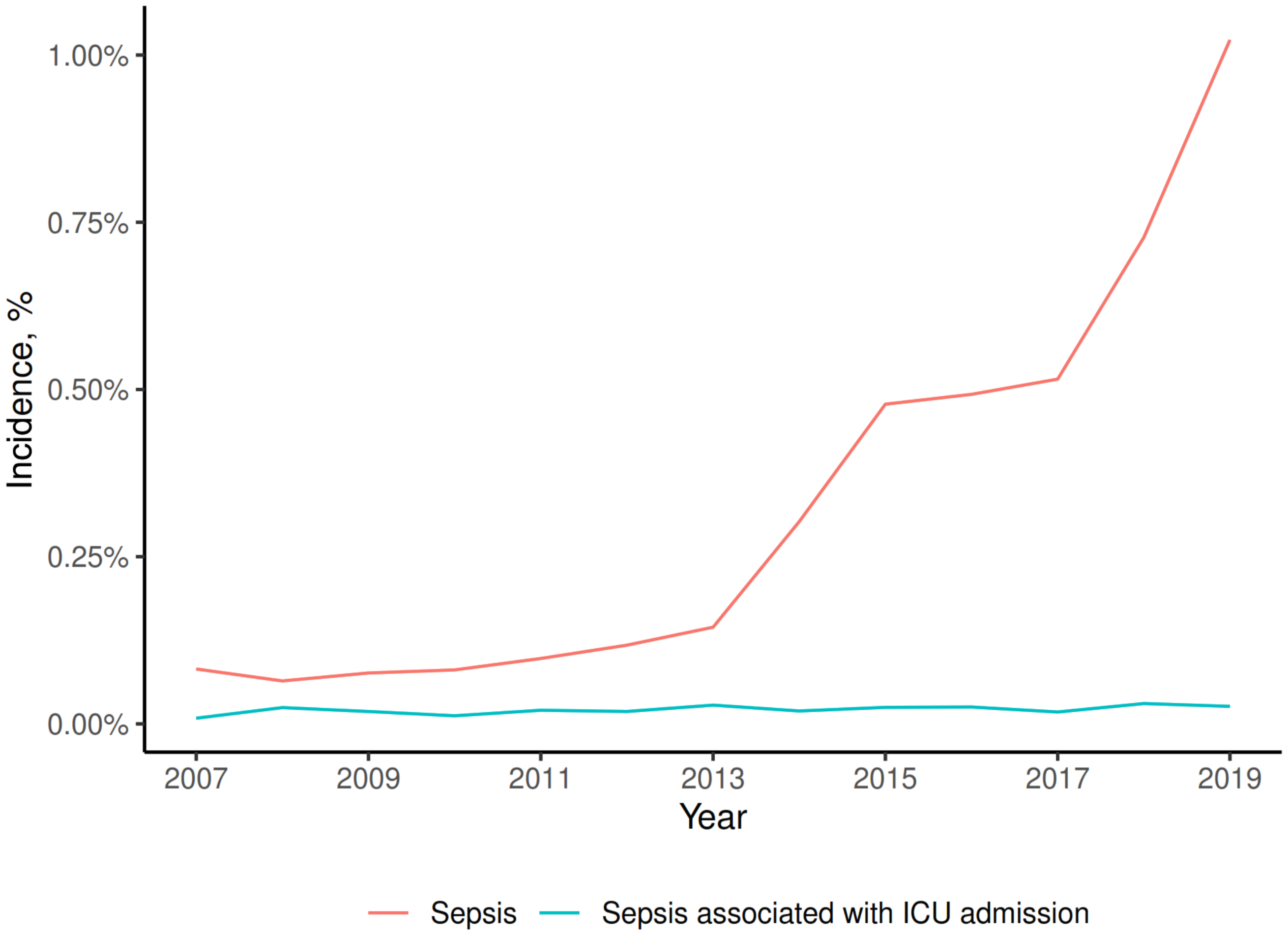

eFigure 3a **Directed Acyclic Graph (DAG) for analysis of whole cohort and preterm births.**

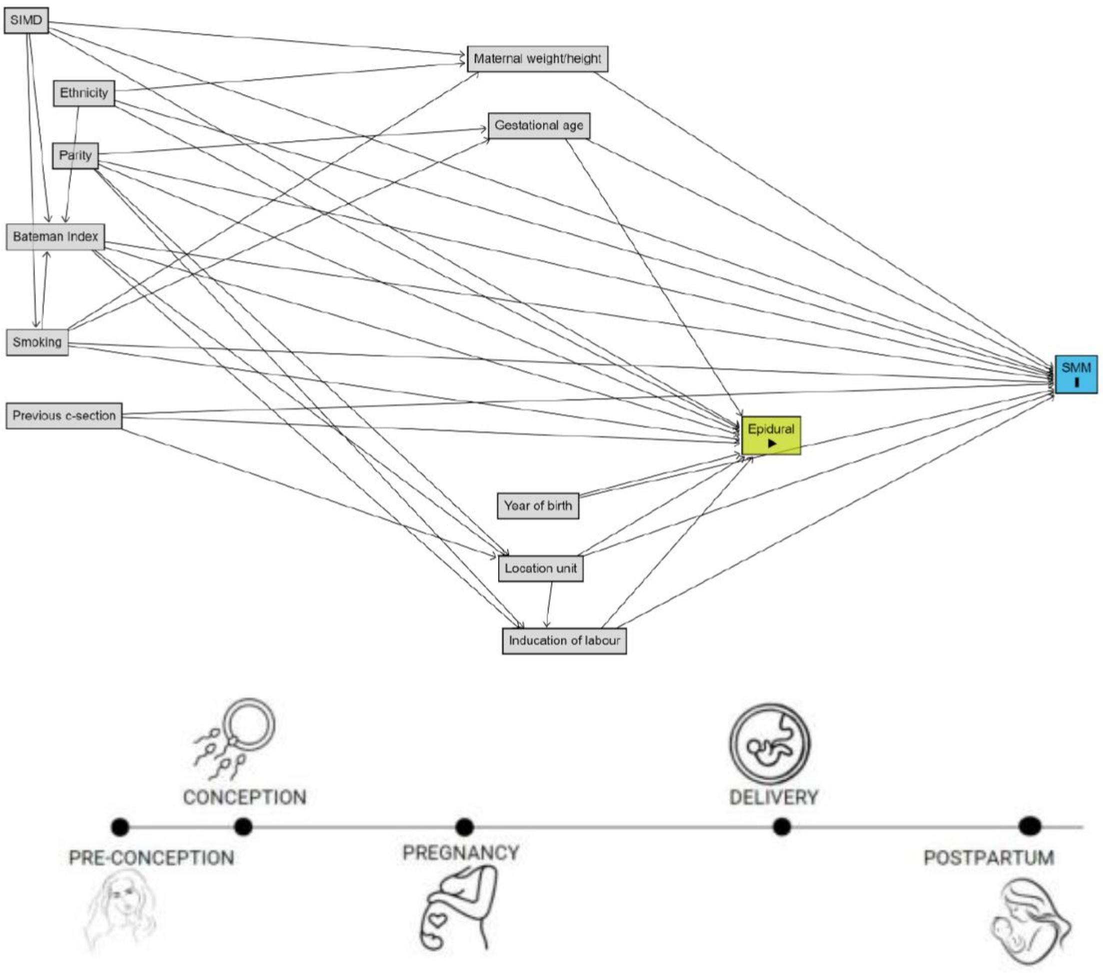

SIMD = Scottish Index of Multiple Deprivation score. Prior caesarean section is included as a component of the Bateman Index if it occurs between 180 days pre-conception to one day before delivery. SMM = Severe Maternal Morbidity. Caesarean sections prior to this time period are included as “previous c-sections”.
Green box = exposure.
Blue box = outcome.

eFigure 3b **Directed Acyclic Graph (DAG) for analyses stratified by "medical indication for epidural"**

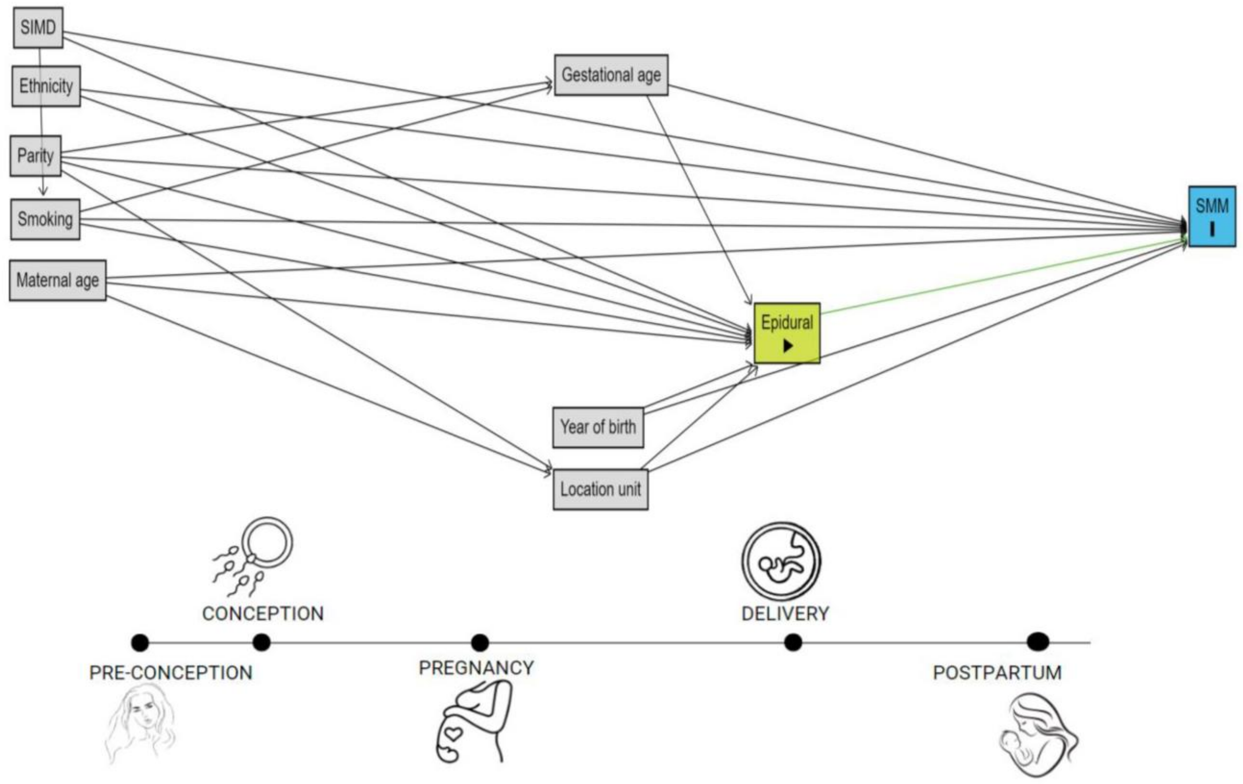

SIMD = Scottish Index of Multiple Deprivation score. SMM = Severe Maternal Morbidity.
Green box = exposure.
Blue box = outcome.

eFigure 4 Adjusted relative risk for all components of SMM compared with not having an epidural (RR=1)

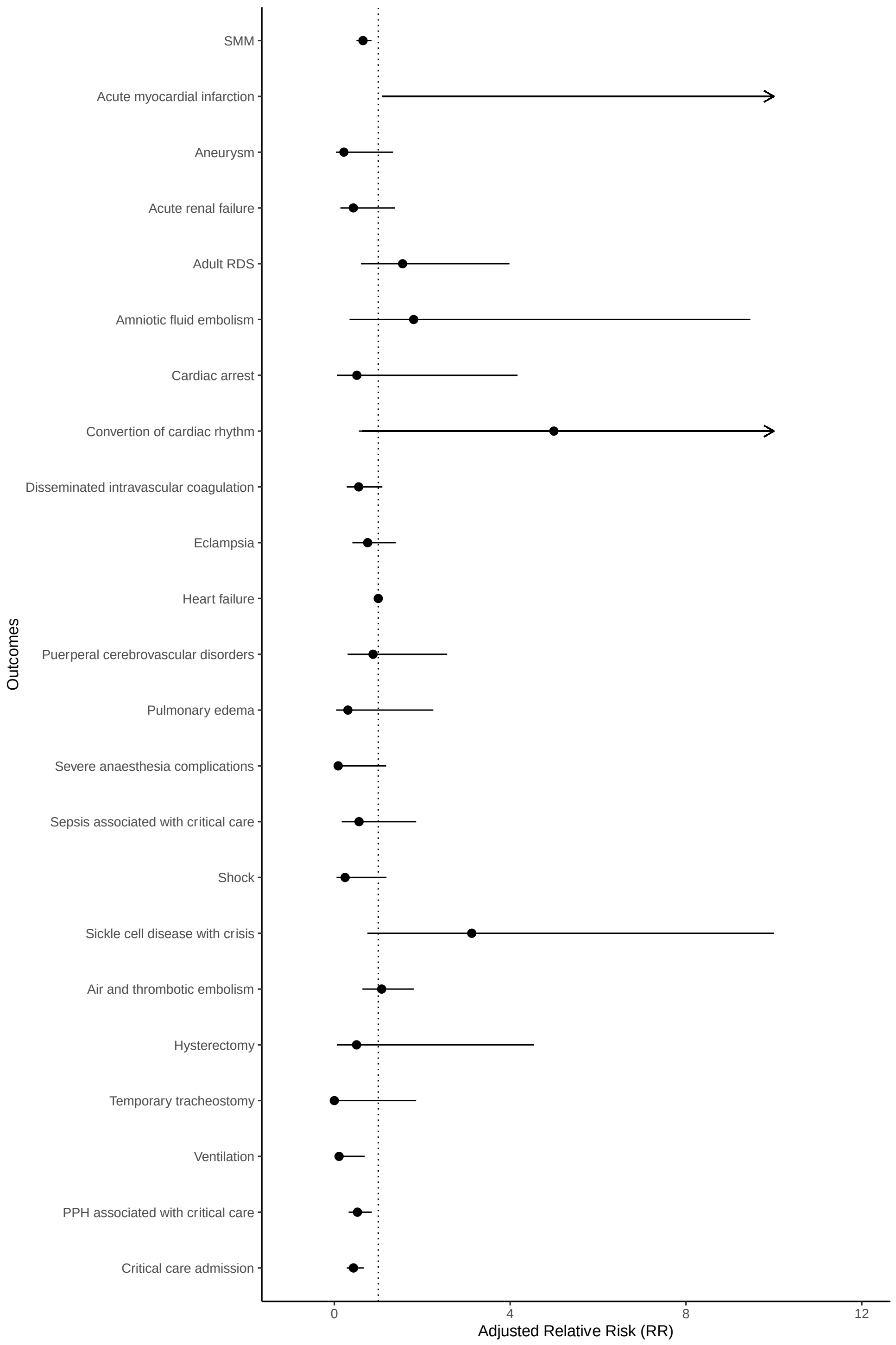

SMM = Severe Maternal Morbidity. PPH = postpartum haemorrhage. Adult RDS - Adult Respiratory Distress Syndrome.
